# Author’s reply

**Published:** 2011

**Authors:** S Manikandan

**Affiliations:** *Assistant Editor, JPP*

Sir,

I thank the author for his interest in and comments[1] on our article.[2] The Postgraduate Corner section hosts a series of small articles on statistics. Many topics are planned in this series which might be published over 10–15 issues. The points relevant to the topic only are discussed. The article commented upon is about data transformation, and a separate one on normal distribution is also planned. Hence all details need not be provided in a single article. More details will appear in articles published later.

The first part of the article tries to explain when one needs to transform data. The objective of this part is not to inform the readers which test should be selected – parametric or nonparametric. So these details are not necessary here.

The author (of the letter) has not supported the first paragraph of point 1 with specific references. Still it is clarified that it is written on “biological parameters” and nature includes diverse biology. The examples using human biological parameters are illustrated since the readers of this journal belong to this field.

It is written in the published article, “This is called normal distribution as most of the biological parameters (such as weight, height and blood sugar) follow it.” The author (of this letter) has already commented on this line in the first paragraph. Again the author is breaking this line as “Most of the biological parameters follow normal distribution” and is trying to argue. This makes the argument out of context (even though what is written might be correct).

Point 2 is acceptable. It is appropriate to specify that only a parametric test needs this assumption.

The published article clearly describes only some simple ways to detect skewness. The intention here is to give a few simple methods and not an exhaustive list as the article is meant for young researchers and not statisticians. Even though the visual observation of a histogram and box and whisker plot is easy, it is unreliable, hence not mentioned. I like to quote Altman from his article published in the British Medical Journal, “Visual inspection of the distribution may suggest whether the assumption of normality is reasonable but (as [Figure 3] suggests) this approach is unreliable.”[3] The fact that normality can be checked by a statistical test is already mentioned in the published article.

The reason for advising data transformation instead of using a nonparametric test is already highlighted in the article – the parametric tests are more robust. If we cannot transform data, then one has to resort to nonparametric tests only.
